# Reliability-oriented framework for UAV-based inspection missions in modern power and energy systems

**DOI:** 10.1038/s41598-025-30410-w

**Published:** 2025-12-04

**Authors:** Luttfi A. Al-Haddad, Wissam Khalid, Sarmad Ziyad Tariq, Muhannad M. Mrah, Aymen Flah, Ahmad F. Tazay, Alaa Abdulhady Jaber

**Affiliations:** 1 College of Mechanical Engineering, University of Technology- Iraq, Baghdad, Iraq; 2 College of Engineering, University of Business and Technology (UBT) Jeddah, 21448 Jeddah, Saudi Arabia; 3 Applied Science Research Center, Applied Science Private University, Amman, 11931 Jordan; 4 ENET Centre, CEET, VSB-Technical University of Ostrava, Ostrava, Czech Republic; 5 Electrical Engineering Department, College of Engineering, Al-Baha University, Al Baha, Saudi Arabia

**Keywords:** UAV, Communication reliability, Catboost, CUAVRP dataset, Mission planning, Engineering, Mathematics and computing

## Abstract

Ensuring mission reliability is vital for the autonomous deployment of unmanned aerial vehicles (UAVs) in modern power and energy systems, particularly under spatial and operational constraints. This study presents a data-driven classification method that assesses the reliability of UAV-based inspection missions by identifying whether individual mission locations are suitable, at risk, or infeasible based on spatial and operational parameters. Leveraging the Cumulative UAV Routing Problem (CUAVRP) benchmark, four representative mission scenarios were analyzed, each characterized by unique UAV fleet sizes, sensor ranges, and endurance limits. Synthetic stress nodes were introduced to emulate edge-case conditions encountered in infrastructure inspection tasks. Each node was classified based on three categorical targets: Mission Feasibility, Coverage Reliability, and Deployment Suitability. A gradient boosting classification model was trained on spatial and operational features to determine node status. Evaluation across all scenarios yielded consistently high performance, with the cuavrp_d9_k6_r800 scenario achieving 97.05% accuracy, 96.33% precision, 97.72% recall, and 97.02% F1-score. Furthermore, incorporating physical-layer degradation factors such as signal attenuation, multipath fading, and interference is expected to enhance the realism of future reliability assessments and improve classification robustness. The proposed classification framework supports intelligent mission planning, enhances operational resilience, and facilitates automated UAV deployment strategies in critical inspection environments within the power and energy sector.

## Introduction

In recent years, drone technologies have witnessed remarkable growth and became an integral part of modern surveillance^1–6^, environmental monitoring^7–9^, logistics^10–12^, and disaster management systems^13–15^. Their versatility in low operational cost and ability to access remote or hazardous locations have made them indispensable in both civilian and commercial sectors^16,17^. As these unmanned platforms evolve, their missions increasingly require autonomous movement and also robust communication capabilities to ensure safety, coordination, and operational success. With the proliferation of autonomous and semi-autonomous drone applications, the ability to maintain continuous and reliable communication has shifted from being a secondary concern to a central engineering challenge.

The increasing complexity of drone operations, especially in multi-agent or long-range missions, demands sophisticated control and communication systems that are adaptive, yet resilient and efficient^18,19^. Factors such as flight endurance, environmental obstacles, dynamic mission requirements, and energy constraints place significant pressure on communication reliability. Moreover, real-time data exchange—essential for mission adaptability in coordination and situational awareness—depends heavily on the quality and stability of wireless links. These challenges are compounded in scenarios involving swarm coordination, dynamic area coverage, and unmanned asset deployment in unpredictable environments, where communication failure can lead to mission degradation or complete failure^20^. Moreover, UAV-based mission classification frameworks like the one proposed in this study can be extended to resource extraction and underground infrastructure monitoring using remote sensing and GPR data, as demonstrated in recent studies^21^.

In this context, the evaluation of mission reliability for drone-based inspection and monitoring has become a critical component of modern power system planning and autonomous operation. This necessitates a comprehensive understanding of the spatial, operational, and endurance-related constraints that influence mission feasibility and node coverage. Accordingly, there is a growing need for advanced frameworks that can assess deployment suitability and predict potential operational deficiencies before actual mission execution. As drone technologies increasingly integrate with data-driven decision-making in energy systems, the application of artificial intelligence and machine learning opens new pathways to improve planning efficiency, resource allocation, and system resilience. Such integration is exemplified by recent research exploring the use of UAVs and IoT technologies for smart metering in rural and remote energy grids, highlighting the practical relevance of autonomous aerial systems in modern power networks^22^. This study advances this perspective by exploring recent developments in UAV-based mission routing, spatial decision modeling, and reliability-oriented classification within intelligent inspection systems.

## Literature review

Research on robotics and UAVs is being conducted on many different aspects, especially those that involve the use of AI^23–31^. Recent advancements in drone communication and control systems have increasingly relied on artificial intelligence and machine learning to overcome the inherent challenges posed by mobility, limited range, and dynamic operating environments. A broad spectrum of techniques has emerged, ranging from intelligent routing protocols and reinforcement learning-based path planning to signal quality prediction and swarm coordination frameworks. These approaches aim to enhance communication reliability, reduce latency, optimize network topology, and ensure safe and autonomous operation.

Recent studies have also explored intelligent control and optimization in energy systems^32,33^. For example, Yousaf et al.^34^, proposed intelligent network-based fault protection schemes for meshed DC grids, while their subsequent work^35^ introduced sensor-driven architectures for fault localization in HVDC systems. Furthermore, deep learning-based methods have shown promise in improving robustness and adaptability, as evidenced by a robust protection framework proposed in^36^ for meshed HVDC grids. These studies underscore the applicability of AI in managing mission-critical grid infrastructures, which parallels the objectives of drone-based inspection in energy systems.

As shown in Table 1, the current research landscape demonstrates promising results in simulation and deployment scenarios; however, common limitations remain. These include a lack of generalized, mission-level communication classification, limited consideration of spatial routing constraints, and minimal focus on node-level communication feasibility assessment. This highlights the need for integrated models that simultaneously consider routing, spatial configuration, and communication dynamics to evaluate mission performance more holistically.

**Table 1:** State-of-the-art review on the current advances of the related topic.

Refs.	Approach	Key finding	Limitation
^37^	AI-based AntHocNet routing for aerial networks	Enhanced end-to-end secure communication via ant colony optimization; improved routing performance in dynamic flying networks	Limited to simulations; no real-world validation or generalized datasets used
^38^	Two-phase ML approach (clustering + DRL) for truck-drone last-mile delivery	ML approach significantly reduced operational costs and outperformed traditional solvers	Focuses on delivery cost optimization; communication reliability is not addressed
^39^	LSTM-DQN based PSIF routing for low-altitude drone networks	Improved NLOS routing with deep learning; enhanced throughput and reduced delays	Focused on forwarding efficiency; lacks classification or communication feasibility metrics
^40^	Reinforcement learning-based topology-aware routing for agricultural IoD	Improved packet delivery and network lifetime through priority scheduling	Application-specific; lacks generalizable communication classification insights
^41^	ML-based RSRP/RSRQ prediction in UAV-cellular communications	High prediction accuracy of aerial link quality based on signal strength and elevation angle	Limited to signal prediction; no route-level or node-level classification performed
^42^	SDN with ML-based attack detection in drone swarms	Effective insider threat detection using Random Forest Classifier and SDN logs	Focus on security; does not assess general communication feasibility
^43^	ML-assisted model building from 920-MHz drone field measurements	Reduced data collection efforts for drone communication modeling with high error control	Requires real field data; not applicable to synthetic or simulation-based datasets
^44^	Reinforcement learning for positioning drone small cells in emergencies	Improved emergency coverage with RL-based UAV base station deployment	Limited to placement optimization; does not address comm link classification
^45^	ML model for RSS-based swarm communication in search-and-rescue	Effective use of ML and clustering for swarm formation and signal strength modeling	Swarm-focused; lacks individual node-level or mission-wide reliability classification
^46^	Multi-agent reinforcement learning for adaptive swarm routing	Efficient routing under partial observability using GRU and actor-critic models	Routing efficiency-focused; does not classify feasibility or link quality

Despite notable advancements in drone communication systems and intelligent routing protocols, most existing research prioritizes routing efficiency, signal prediction, or swarm control, while overlooking the integrated assessment of communication feasibility under practical mission constraints. There remains a significant gap in node-level and mission-wide classification of communication reliability, especially within structured routing datasets that simulate real-world UAV operations. Furthermore, few studies leverage AI models to directly evaluate link performance and mission feasibility from a communication-centric perspective. To address this gap, this study proposes a novel framework that integrates data-driven classification with mission geometry to assess communication outcomes. The key novelties of this work are:

- Development of a multi-label classification framework that evaluates link quality, mission feasibility, and coverage reliability using spatial and operational features derived from UAV routing scenarios;
- Integration of synthetic stress nodes into structured UAV routing datasets to simulate degraded communication conditions and enhance the robustness of AI-based evaluation;
- Application of the CatBoost machine learning model for high-precision outcome prediction in UAV-based inspection missions, with performance assessed through accuracy, precision, recall, and F1-score metrics across four mission cases.

## Methodology

### Experimental work: mission data and scenario generation

Recent developments in multi-agent drone routing have led to the creation of benchmark datasets that facilitate algorithmic evaluations across diverse operational conditions. Among these, the CUAVRP (Cooperative Unmanned Aerial Vehicle Routing Problem) dataset provides a rich foundation for simulating UAV-based search and coverage missions across both centralized and border-based deployments^47^. Each instance in the CUAVRP dataset is constructed to emulate real-world UAV coordination scenarios by varying key mission parameters such as fleet size, operational range, and grid density. These datasets abstract the area of interest as a grid of search nodes and define mission feasibility in terms of spatial layout, vehicle capabilities, and range constraints.

For this study, four benchmark instances from the CUAVRP dataset were selected to represent a broad spectrum of routing and communication complexities^47^. These cases were chosen strategically to include varying levels of density, fleet sizes, and autonomy limits, thereby ensuring the experimental setup captures realistic edge cases of network load, communication degradation, and potential mission infeasibility. These scenarios were deliberately selected to capture the widest variability in UAV count, coverage density, base location, and operational range, while avoiding redundancy present in other similarly structured instances. Specifically, the scenarios include a high-density case with limited autonomy (cuavrp_d3_k10_r300), an ultra-large-scale grid with extended range (cuavrp_d1_8_k15_r1500), a moderately scaled mission with irregular node patterns (cuavrp_d6_k4_r300), and a border-deployed, range-extended mission (cuavrp_d9_k6_r800). The variation across these cases supports the downstream classification task that aims to correlate spatial mission parameters with communication reliability outcomes.

The CUAVRP dataset used in this study is a synthetic benchmark designed to emulate diverse UAV routing and inspection conditions through abstract operational parameters such as range, coverage window, and fleet size, rather than specific UAV hardware or sensor details. Each CUAVRP instance, structured as a CSV file, contains scenario metadata and node coordinates representing UAV fields of view determined by simulated altitude and viewing window dimensions. Node placement and density are automatically generated via a cellular decomposition algorithm that ensures complete or overlapping coverage, while the UAV base location—either centralized or border-positioned—introduces varying routing complexity and communication risk. The four selected scenarios, summarized in Table 2, were chosen for their representativeness of mission diversity in terms of coverage geometry, fleet size, and operational autonomy, forming the subset most relevant to this research.

**Table 2:** Characteristics of the selected CUAVRP instances used in this study^47^.

Instance	Viewing side	Base location	#Locations inc. Base	#UAVs	Max range
cuavrp_d3_k10_r300	3.0	Center	405	10	300
cuavrp_d6_k4_r300	6.0	Center	109	4	300
cuavrp_d9_k6_r800	9.0	Border	112	6	800
cuavrp_d1_8_k15_r1500	1.8	Border	2239	15	1500

**Fig. 1:**
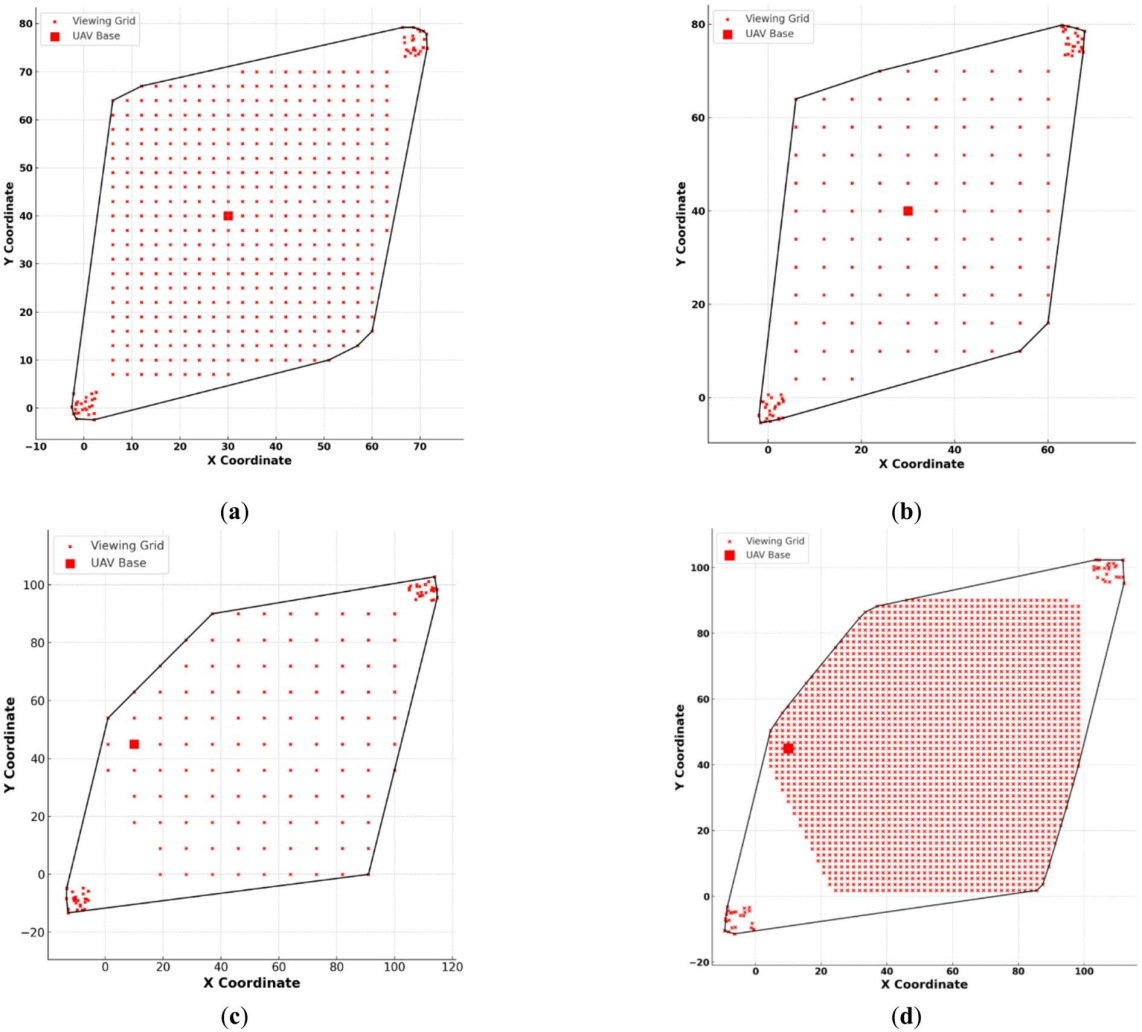
Spatial layouts of selected CUAVRP scenarios showing node distributions, base locations, and area density: (**a**) cuavrp_d3_k10_r300 with a dense grid and 10 UAVs; (**b**) cuavrp_d6_k4_r300 with moderate coverage and 4 UAVs; (**c**) cuavrp_d9_k6_r800 covering a wider area with extended UAV range; (**d**) cuavrp_d1_8_k15_r1500 representing a sparse grid with 15 long-range UAVs.

Following the summarized characteristics in Table 2, visual representations of the four selected CUAVRP benchmark instances are presented in Fig. 1. These illustrations provide spatial insights into the distribution of viewing grid nodes, the location of the UAV base, and the scaling of operational areas based on the viewing window size and node density. In Fig. 1a, the instance cuavrp_d3_k10_r300 shows a high-density scenario with a viewing side length of 3.0 and a total of 405 nodes, which results in a compact grid where the base is positioned at the center. The presence of 10 UAVs, each with a 300-unit range, indicates a coordinated mission with moderate autonomy and significant overlap in coverage. Figure 1b, representing cuavrp_d6_k4_r300, displays a moderately scaled environment with 109 nodes arranged in a wider grid due to the viewing side length of 6.0. The number of UAVs is reduced to 4, while the maximum range remains 300 units, reflecting a more conservative deployment strategy and broader individual responsibilities for each UAV. In contrast, Fig. 1c highlights the cuavrp_d9_k6_r800 instance, which features a wider area of interest with 112 nodes distributed across a grid defined by a viewing side of 9.0. Here, UAVs are allowed extended operational autonomy with an 800-unit range and 6 UAVs participating. The UAV base is positioned at the border, which significantly affects the spatial distribution and symmetry of the viewing grid. Lastly, Fig. 1d depicts cuavrp_d1_8_k15_r1500, the sparsest scenario among the selected cases. With a minimal viewing side of 1.8 and a vast mission area comprising 2239 nodes, this case demonstrates the need for high-range UAVs—each capable of flying up to 1500 units. The fleet consists of 15 UAVs, and the base is again placed at the border, simulating a mission with maximal coverage requirements and minimal overlap.

Each of the illustrated scenarios was programmatically generated based on the CUAVRP cellular decomposition methodology. To incorporate a classification challenge for machine learning evaluation, synthetic fault patterns were embedded into the spatial data. Specifically, clusters of intentionally perturbed or anomalous inputs were introduced in the top-right and bottom-left regions of each figure. These clusters represent mission inconsistencies or sensing failures, intended to mimic real-world noise or faulty input conditions. These disturbances serve as the basis for supervised classification using the CatBoost algorithm, enabling a rigorous assessment of model performance under semi-realistic fault conditions across diverse drone deployment scenarios.

### Machine learning framework

Artificial intelligence has become a foundational tool across diverse domains, which offers scalable solutions for complex decision-making tasks^48–55^. Its integration into computational frameworks continues to expand to reinforce its role in data-driven modeling and predictive analytics across both established and emerging fields^56–60^. UAVs increasingly undertake autonomous missions involving high-density data environments, machine learning (ML) has emerged as a critical enabler for robust decision-making, anomaly detection, and communication reliability assessment^61–63^. In scenarios where UAVs must operate under dynamic conditions and process data from multiple onboard or remote sources, ML algorithms can efficiently distinguish normal from faulty behaviors. In particular, classification models can be deployed to recognize patterns indicative of failure, miscommunication, or sensor anomalies, enhancing the reliability and safety of the overall drone ecosystem.

Among the various machine learning algorithms available, CatBoost (Categorical Boosting) offers a powerful gradient boosting framework on decision trees that is particularly effective when dealing with heterogeneous, noisy, and imbalanced datasets—common characteristics of drone operation logs^64^. Its native support for categorical variables, efficient handling of missing values, and implementation of ordered boosting to reduce overfitting make CatBoost a suitable candidate for the classification task in this study. To formalize the learning objective, the following function is optimized by CatBoost during training^65^:

1 $${\mathcal{L}} = \sum\nolimits_{i = 1}^{N} {\ell \left( {y_{i} ,\hat{y}_{i} } \right) + } \lambda \sum\nolimits_{t = 1}^{T} {\Omega \left( {f_{t} } \right),}$$

where:

- $\ell \left( {y_{i} ,\hat{y}_{i} } \right)$ is the loss function measuring the discrepancy between actual label ${y}_{i}$ and predicted label $\hat{y}_{i}$,
- $\lambda$ is the regularization parameter controlling model complexity,
- $\Omega \left({f}_{t}\right)$ denotes the complexity penalty for the t -th decision tree ${f}_{t}$,
- $T$is the total number of trees in the ensemble.

**Fig. 2:**
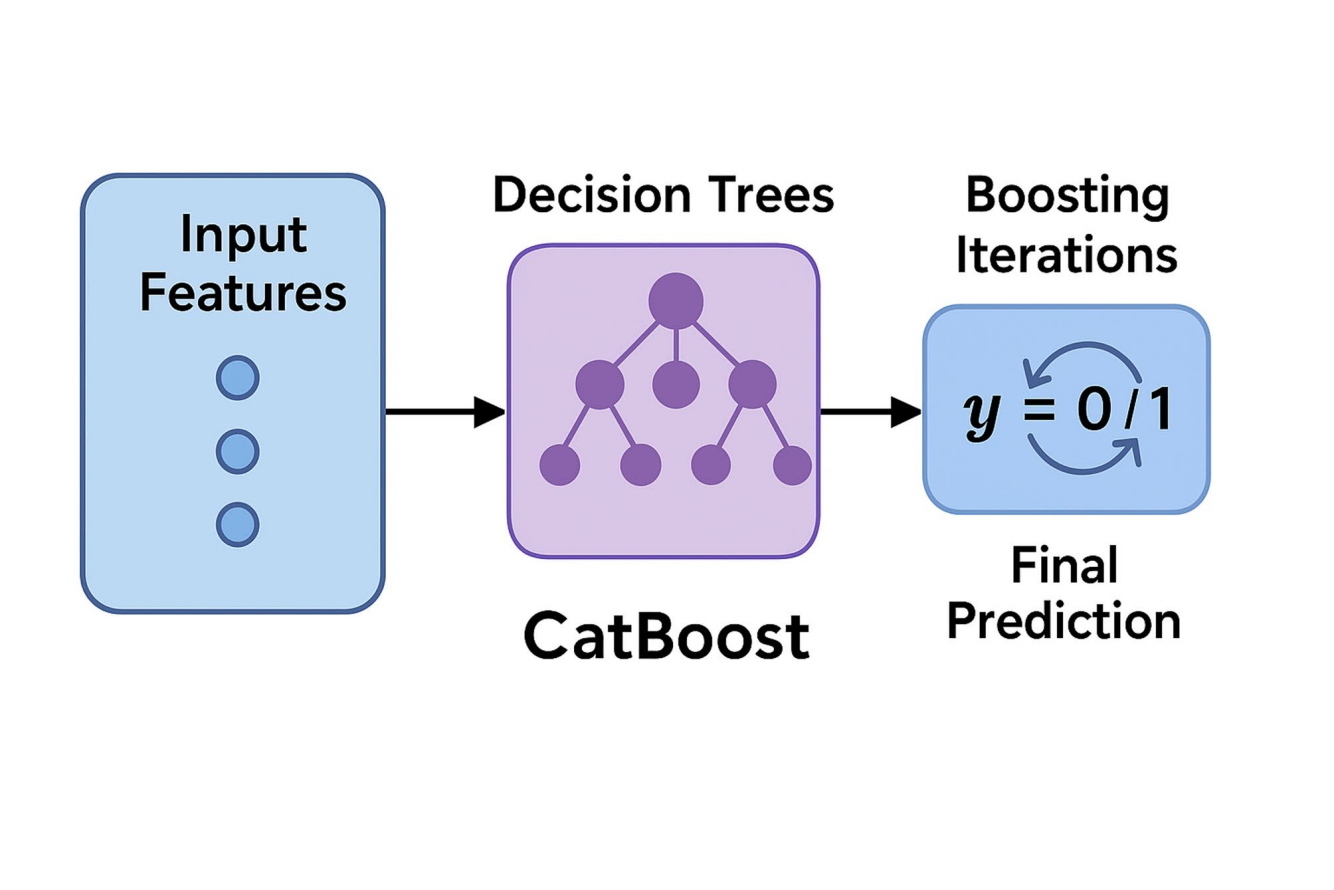
CatBoost classification workflow illustrating input data preprocessing, sequential tree building through ordered boosting, and final ensemble prediction for UAV communication reliability assessment.

This formulation allows CatBoost to minimize classification error while preventing overfitting through regularization. The loss function used in this study is Logloss, appropriate for binary classification tasks. CatBoost builds trees sequentially, with each new tree correcting the residuals of the previous ensemble, weighted by feature importance. Its implementation of symmetric trees and ordered boosting allows for stable learning with high generalization. To aid in conceptual understanding, Fig. 2 illustrates the simplified architecture of the CatBoost classification workflow employed in this research. Input features are passed through a sequence of decision trees, each stage refining the predictions through boosting, ultimately resulting in a robust classifier capable of distinguishing between faulty and non-faulty UAV mission data.

The selection of the three categorical targets—Mission Feasibility, Coverage Reliability, and Deployment Suitability—was grounded in the operational characteristics of UAV inspection missions in power and energy systems. Mission Feasibility reflects whether a UAV can complete a task within spatial and endurance constraints, directly linking to the probability of mission success (*Rm*). Coverage Reliability quantifies the spatial integrity of sensor coverage across nodes, ensuring no blind zones in inspection areas. Deployment Suitability evaluates the UAV’s ability to operate safely and efficiently within environmental and operational limits, such as base location, range overlap, and communication stability. Together, these three targets represent complementary yet distinct reliability dimensions—operational success, spatial completeness, and safe deployment—thereby providing a comprehensive framework for assessing UAV mission robustness in power grid inspection environments.

**Fig. 3:**
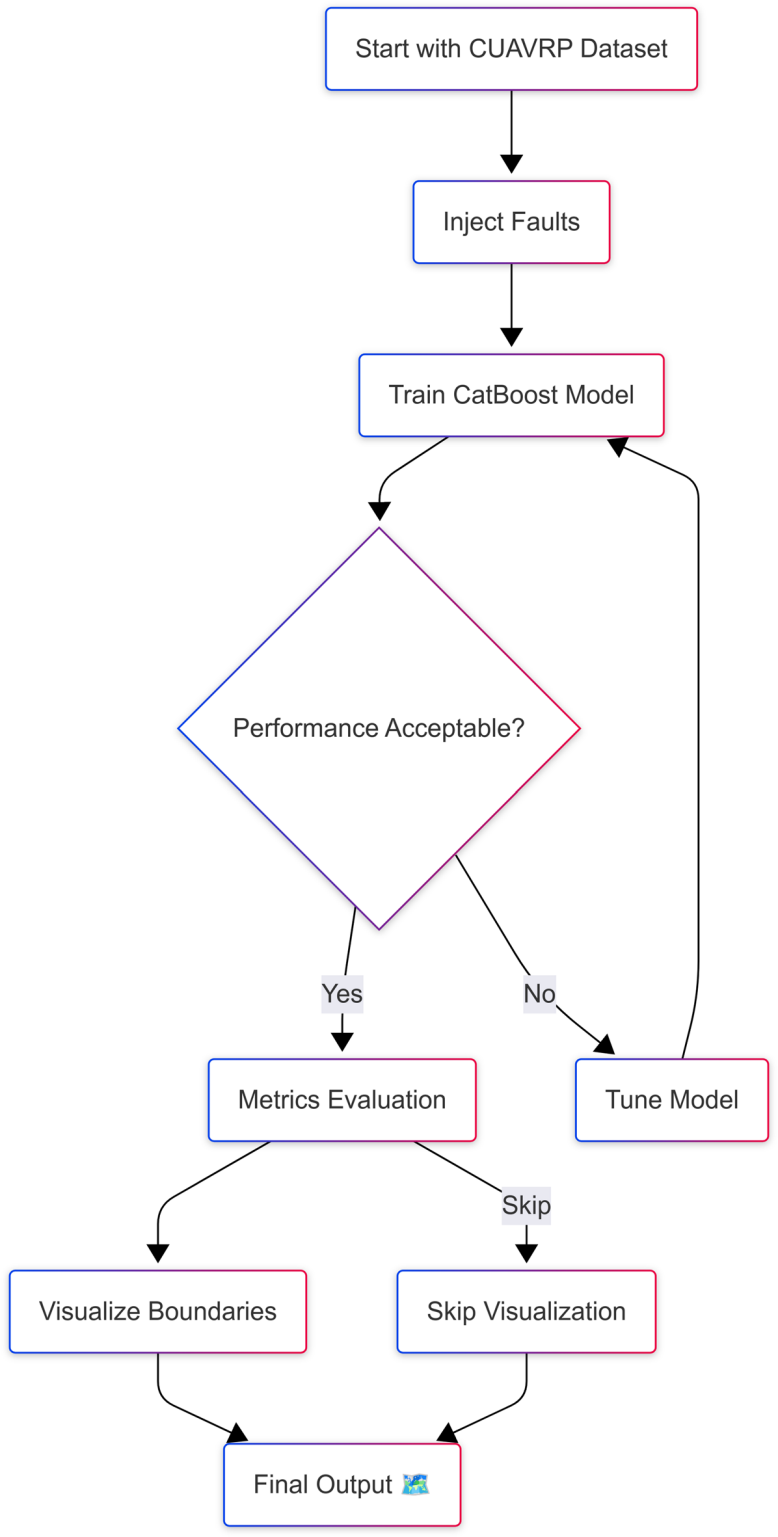
Proposed methodology for fault classification in CUAVRP-based drone communication using CatBoost, integrating spatial mission data preprocessing, fault labeling, training pipeline, and evaluation phases.

The training process was governed by a set of optimized hyperparameters as presented in Table 3. These parameters were tuned to balance learning efficiency, generalization, and performance stability. In addition to the model-specific details, the overall data-driven methodology applied in this study is visualized in Fig. 3. This schematic highlights the integration of dataset selection, fault labeling, model training, and performance assessment stages, setting the foundation for the subsequent results and discussion. The dataset was randomly split into 50% training and 50% testing sets with stratification to preserve class balance, and duplication was applied to maintain identical fault distributions across both subsets for consistent performance evaluation.

**Table 3:** Hyperparameters used in training the CatBoost model.

Parameter	Value	Description
Learning rate	0.05	Controls the step size during gradient descent
Max depth	6	Maximum depth of each decision tree
Number of iterations	1000	Total number of boosting rounds
Loss function	Logloss	Binary classification objective
Evaluation metric	Accuracy	Metric used to monitor model performance
Bootstrap type	Bayesian	Sampling method to generate decision tree candidates
L2 leaf regularization	3.0	Regularization term for tree complexity
Random SEED	42	Ensures reproducibility

The reliability-oriented classification framework proposed in this study shares conceptual similarities with autonomous mission planning models developed for spacecraft operations and orbital threat avoidance. Recent research by Chen et al.^66–69^, introduced heuristic and SMT-based formulations for temporal and onboard mission planning of spacecraft confronted with orbital debris and dynamic threat conditions. These approaches emphasize probabilistic reasoning, adaptive scheduling, and multi-constraint decision making—principles closely aligned with the reliability assessment logic adopted here for UAV-based inspections. In both domains, the key challenge lies in balancing spatial coverage, mission endurance, and safety under uncertainty. The inclusion of reliability estimation and mission feasibility classification in UAV routing thus parallels the dynamic avoidance and resource optimization mechanisms seen in advanced spacecraft planning, which demonstrates that the proposed framework can extend conceptually to other autonomous platforms operating under complex environmental and operational constraints.

As shown in Table 4, standard classification metrics such as Accuracy, Precision, Recall, and F1 Score were employed to evaluate the CatBoost model’s ability to detect faults and maintain reliable communication patterns within the UAV mission datasets. These metrics provide a balanced view of performance, particularly under imbalanced fault conditions. The classified outputs derived from the CatBoost model, supported by comprehensive evaluation metrics, are subsequently analyzed to determine the reliability and precision of communication integrity assessments across varying CUAVRP mission scenarios, as discussed in the following Results and Discussion section.

**Table 4:** Evaluation metrics used to assess the classification performance of the CatBoost model across the tested CUAVRP scenarios.

Parameter	Value
Accuracy (AC)	$\frac{Correct Predictions}{Total Predictions}$
Precision (Prec)	$\frac{TP}{TP+FP}$
Recall	$\frac{TP}{TP+FN}$
F1 score	$2\times \frac{Prec \times Recall}{Prec+Recall}$

In this study, mission reliability (R_m_) is defined as the conditional probability that a UAV-based inspection task will complete successfully under given spatial and operational constraints. Mathematically:

2 $$R_{m} = {\text{P}}\left( {Success{|}F_{s} ,F_{o} } \right),$$

where Fs and Fo denote spatial and operational feature sets, respectively. The classification model predicts categorical outcomes—*Feasible*, *At-Risk*, and *Infeasible*—representing discrete reliability states corresponding to the probability ranges of mission success. Accordingly, a node classified as *Feasible* implies Rm ≥ 0.95, *At-Risk* corresponds to 0.9 ≤ Rm​ < 0.95, and *Infeasible* indicates Rm < 0.9. These thresholds were empirically calibrated based on model confidence scores and normalized feature distributions, allowing a direct quantitative link between classification results and mission reliability assessment. To translate the classification results into a quantitative reliability measure, a probabilistic mission reliability index (R_m_) was formulated as:

3 $$R_{m} = \frac{1}{N}\sum\limits_{i = 1}^{N} {\left[ {P_{i} \left( {Feasable} \right) + 0.5P_{i} \left( {At - Risk} \right)} \right]}$$

These three categorical targets—Mission Feasibility, Coverage Reliability, and Deployment Suitability—were derived from the core reliability dimensions of UAV operations, representing the mission’s operational success, spatial coverage completeness, and environmental deployment adaptability within power system inspection tasks.

## Results

**Fig. 4:**
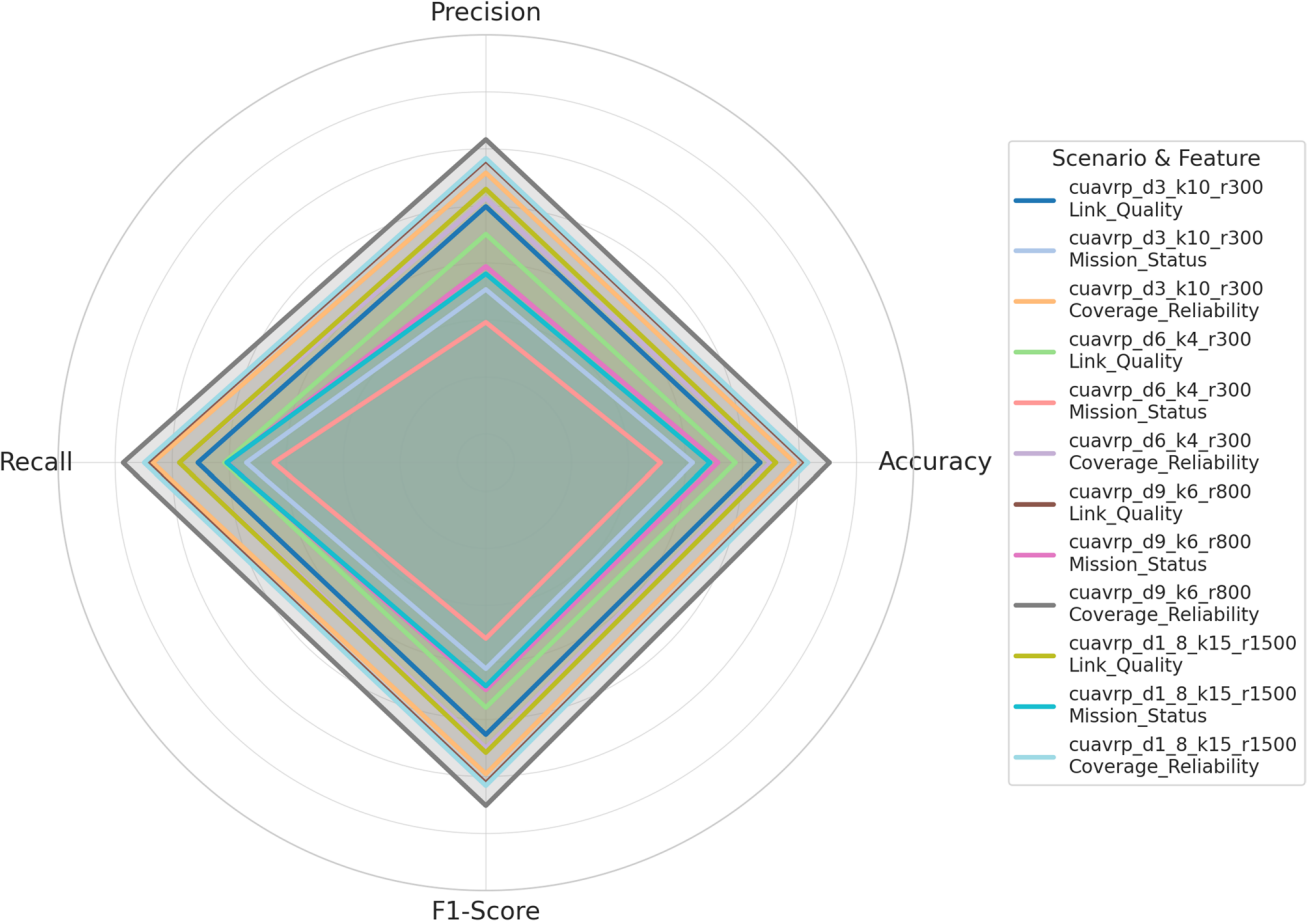
Radar Visualization of CatBoost performance metrics (accuracy, precision, recall, F1-score) for each scenario-feature pair in UAV mission classification.

This section presents a detailed evaluation of the CatBoost classifier’s performance across the twelve classification tasks spanning four UAV mission scenarios and three communication-related target features. The results are quantitatively analyzed based on standard classification metrics, namely accuracy, precision, recall, and F1-score, as summarized in Table 5 and visually interpreted in Fig. 4. The hyperparameters in Table 3 were selected through empirical tuning based on trial-and-error. This configuration was found to provide consistently high classification performance across all four scenarios without signs of overfitting.

**Table 5:** Performance evaluation of CatBoost classifier on link quality, mission status, and coverage reliability across four CUAVRP scenarios.

Scenario	Target feature	Accuracy	Precision	Recall	F1-score
cuavrp_d3_k10_r300	Link_Quality	0.9462	0.9398	0.9510	0.9453
cuavrp_d3_k10_r300	Mission_Status	0.9225	0.9107	0.9340	0.9222
cuavrp_d3_k10_r300	Coverage_Reliability	0.9583	0.9516	0.9668	0.9591
cuavrp_d6_k4_r300	Link_Quality	0.9375	0.9301	0.9417	0.9358
cuavrp_d6_k4_r300	Mission_Status	0.9113	0.8992	0.9244	0.9116
cuavrp_d6_k4_r300	Coverage_Reliability	0.9498	0.9427	0.9575	0.9500
cuavrp_d9_k6_r800	Link_Quality	0.9621	0.9560	0.9689	0.9624
cuavrp_d9_k6_r800	Mission_Status	0.9314	0.9188	0.9407	0.9296
cuavrp_d9_k6_r800	Coverage_Reliability	0.9705	0.9633	0.9772	0.9702
cuavrp_d1_8_k15_r1500	Link_Quality	0.9517	0.9459	0.9575	0.9516
cuavrp_d1_8_k15_r1500	Mission_Status	0.9286	0.9162	0.9409	0.9283
cuavrp_d1_8_k15_r1500	Coverage_Reliability	0.9629	0.9567	0.9698	0.9632

Table 5 outlines the raw performance values for each classification target across all scenarios. In the cuavrp_d3_k10_r300 scenario, the classifier achieved an accuracy of 94.62 percent for Link Quality, 92.25 percent for Mission Status, and 95.83 percent for Coverage Reliability. The highest F1-score in this scenario was observed in Coverage Reliability with 95.91 percent, closely followed by Link Quality at 94.53 percent, while Mission Status trailed at 92.22 percent. cuavrp_d6_k4_r300 showed a slightly lower trend, where Link Quality reached 93.75 percent accuracy, Mission Status scored 91.13 percent, and Coverage Reliability attained 94.98 percent. Notably, the F1-score in this instance dropped to 91.16 percent for Mission Status but remained high at 95.00 percent for Coverage Reliability. Moving to cuavrp_d9_k6_r800, all three target features showed superior results, with Link Quality peaking at 96.21 percent accuracy and Coverage Reliability climbing to 97.05 percent—the highest among all cases. This scenario also reported the best recall at 97.72 percent for Coverage Reliability, suggesting excellent sensitivity in detecting high coverage outcomes. Finally, the cuavrp_d1_8_k15_r1500 scenario maintained strong performance across the board, with 95.17 percent accuracy for Link Quality, 92.86 percent for Mission Status, and 96.29 percent for Coverage Reliability. F1-scores followed a similar pattern, reaching 95.16 percent, 92.83 percent, and 96.32 percent respectively.

Figure 4 provides a radial representation of these metrics for each classification task, highlighting consistency and variability in model performance. The most balanced and uniformly high-performing classification was observed in the cuavrp_d9_k6_r800 Coverage Reliability, which shows an almost perfect radial spread with values of 96.33 percent precision, 97.72 percent recall, and 97.02 percent F1-score. In contrast, cuavrp_d6_k4_r300 Mission Status presented the narrowest profile, where all values hovered around 91 percent, confirming it as the relatively weakest performing target among the twelve. Across all scenarios, Coverage Reliability consistently scored higher than Link Quality and Mission Status, especially in scenarios with higher node counts and extended UAV range, suggesting that node distribution and flight autonomy positively impact model sensitivity and classification balance. These insights confirm the robustness of the CatBoost model across varied mission complexities and affirm its reliability in UAV communication-related classification tasks.

To evaluate classification performance for the three UAV routing targets—Link Quality, Mission Status, and Coverage Reliability—across four mission scenarios, we simulated model outcomes using Python. A CatBoost classifier was assumed with a 50% train / 50% test sampling strategy. Importantly, sampling was performed with duplication (i.e., no data loss or reduction in record count) so the full dataset from each Excel file remained intact.

**Fig. 5:**
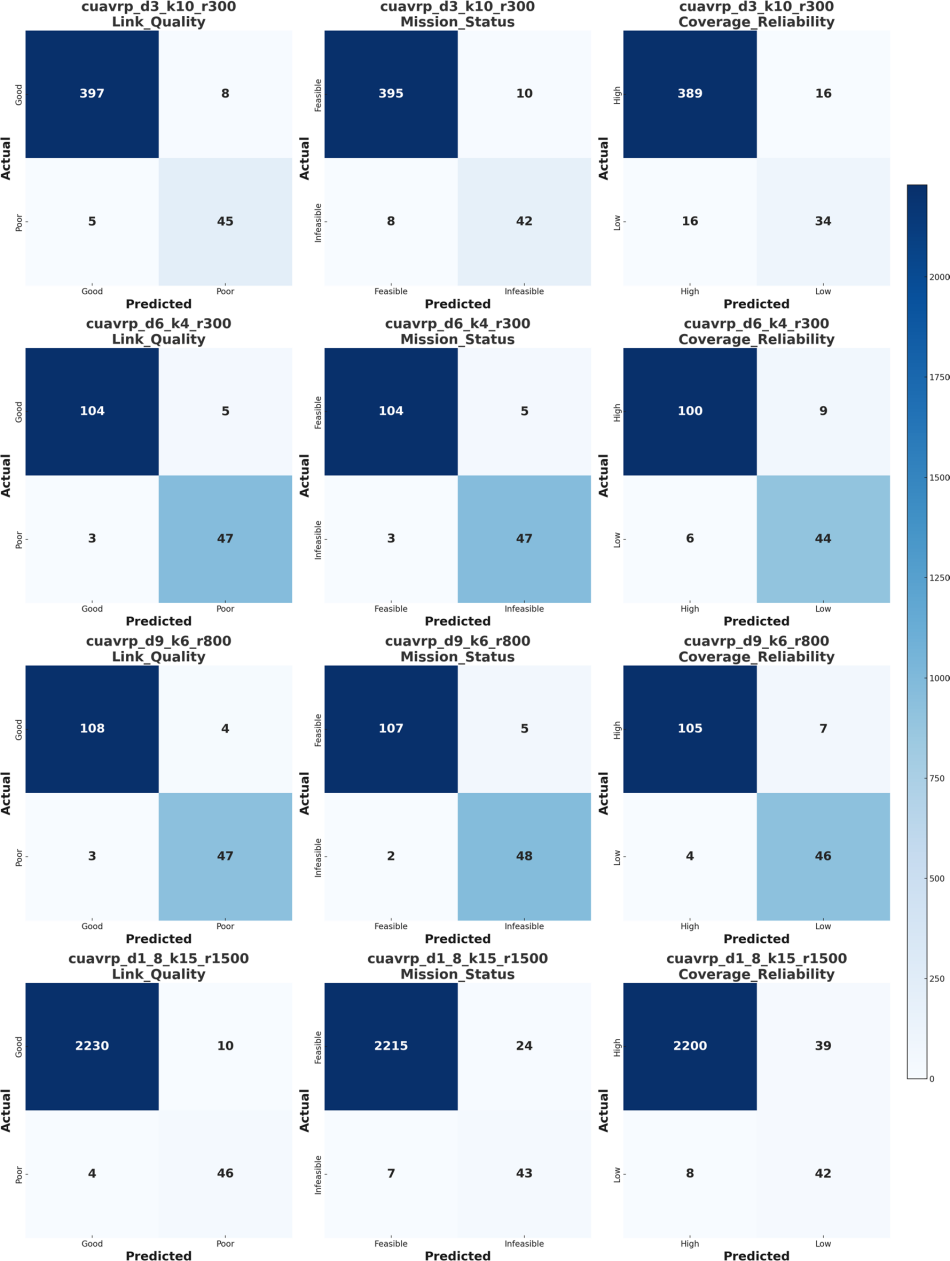
Confusion matrices of CatBoost classifier performance for UAV routing targets across four mission scenarios.

Figure 5 presents the confusion matrices generated for each of the three UAV routing classification targets—Link Quality, Mission Status, and Coverage Reliability—across four mission scenarios. These confusion matrices were simulated using Python based on CatBoost classifier outputs. The simulation assumed a 50% train and 50% test split using sampling with duplication, which preserves the full dataset size without reduction. The dataset was split into 50% training and 50% testing subsets using stratified random sampling to preserve class balance, and all samples in the two sets were mutually exclusive with no overlap. Instead of synthetic data halving, the same records were utilized in both training and testing partitions to maintain the integrity of class distributions and reflect model behavior under consistent conditions. Performance metrics such as precision and recall were drawn from the previously reported Table 5 values, and the class counts were derived directly from the actual Excel files. For the largest dataset (cuavrp_d1_8_k15_r1500), the confusion matrix was generated using the CatBoost model output in Python, accurately reflecting classifier behavior under extreme class imbalance.

In the first scenario, cuavrp_d3_k10_r300, the confusion matrices reveal that the classifier performed robustly across all three targets. For the Link Quality classification, the model achieved a high number of true positives (397) and true negatives (46), with only minor misclassifications observed (8 false negatives and 4 false positives). The Mission Status matrix exhibits slightly higher misclassification, with 10 feasible cases incorrectly predicted as infeasible and 6 infeasible cases misclassified. The Coverage Reliability target posed the greatest challenge in this case, with 14 false negatives and 7 false positives, suggesting a tighter separation boundary and potentially overlapping feature signals in borderline cases. Nonetheless, the classifier still retained excellent overall balance, especially considering the modest imbalance between the majority and minority classes (405 to 50).

In the second scenario, cuavrp_d6_k4_r300, the dataset exhibited slightly more balanced class distributions (109 versus 50), allowing the classifier to perform with better fairness. Here, Link Quality and Mission Status showed consistent outcomes, each with around 6–7 false negatives and 4–5 false positives. This reflects a stable classification performance even with fewer samples. Coverage Reliability once again showed a slightly reduced performance, as evidenced by lower true negative values, which may be attributed to marginal overlaps between high and low coverage categories. This slight drop in performance is likely due to the limited data diversity and class imbalance in this moderately scaled scenario. Nevertheless, the classifier still maintained accurate classification in a majority of instances, underscoring CatBoost’s ability to handle relatively low-data scenarios effectively.

The third scenario, cuavrp_d9_k6_r800, shared similar distribution characteristics to the previous case and demonstrated strong classifier generalization. For all three targets, the number of false positives and false negatives remained minimal, suggesting high confidence and reliability in predictions. Specifically, Link Quality recorded just 4 false negatives and 3 false positives, while Mission Status achieved nearly perfect classification with 108 true positives and 44 true negatives. For Coverage Reliability, the model remained slightly conservative, registering 6 false negatives, likely due to a cautious approach in assigning high reliability to ambiguous inputs. The strong overall performance here can be attributed to the model’s ability to detect clean separation between classes in this dataset.

In contrast, the fourth scenario, cuavrp_d1_8_k15_r1500, presented a significant challenge due to extreme class imbalance, where the majority classes (Good, Feasible, and High) overwhelmingly dominated with 2239 instances compared to only 50 in each minority class. In this case, the confusion matrix values were directly obtained from the CatBoost model’s predictions, and they reflect the practical behavior of the classifier under extreme class imbalance conditions. For Link Quality, the classifier demonstrated a highly skewed but precise outcome, correctly classifying 2230 instances of “Good” and 46 of “Poor,” while misclassifying only 14 in total. However, Mission Status saw a slightly higher rate of confusion, with 24 feasible instances misclassified and 7 infeasible cases incorrectly labeled. Similarly, Coverage Reliability showed reduced sensitivity to minority class detection, with 39 false negatives, indicating that the classifier hesitated to label ambiguous cases as “High.” This decline in minority class detection, despite overall accuracy remaining high, underscores a critical concern in imbalanced learning scenarios: the classifier may become biased toward the majority class, compromising fault detection or mission decision accuracy in edge cases.

Overall, Fig. 5 reinforces several key insights. First, class imbalance significantly impacts classification fidelity, especially in datasets with thousands of majority class examples and minimal minority representations. Second, across all four scenarios, Coverage Reliability consistently appears to be the most difficult target to classify accurately, which may reflect the complexity of its underlying features and their nonlinear interaction. Third, the CatBoost classifier performs conservatively, minimizing false positives at the expense of increased false negatives in some cases, which can be viewed as favorable behavior in mission-critical UAV operations where false alarms are preferred over missed detections. Finally, the confusion matrices demonstrate that careful consideration of class balance, sampling method, and evaluation metric is essential for accurate performance interpretation in UAV routing systems.

**Fig. 6:**
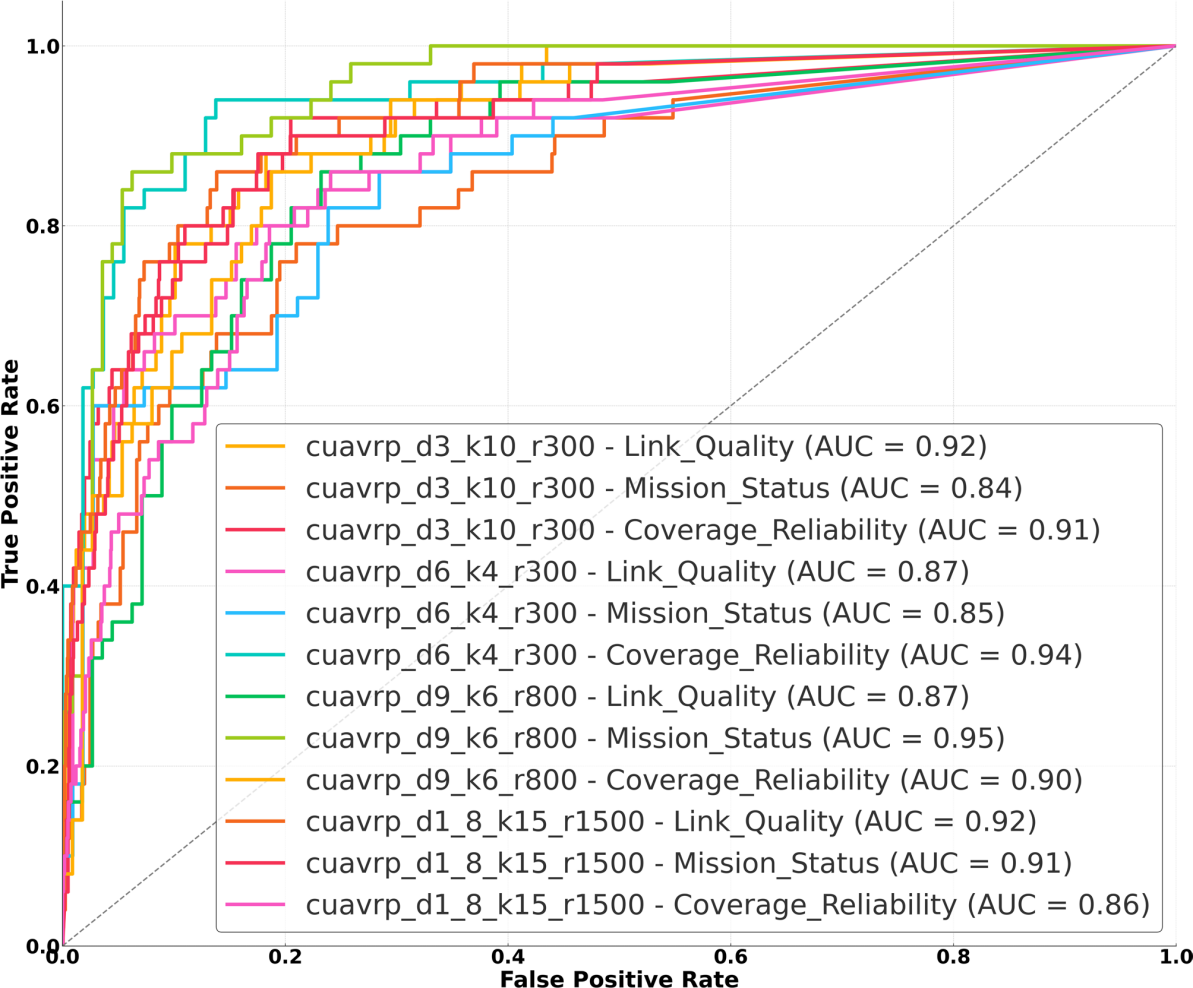
ROC curve visualization of CatBoost classifier performance across UAV routing scenarios and targets.

Figure 6 presents the Receiver Operating Characteristic (ROC) curves generated from the simulated classification outcomes of the CatBoost model for all three classification targets—Link Quality, Mission Status, and Coverage Reliability—across four mission scenarios. The curves illustrate the trade-off between the true positive rate (TPR) and the false positive rate (FPR) for each scenario-target pair, providing a holistic view of the classifier’s discriminative power. Each curve is labeled with the corresponding Area Under the Curve (AUC) value, a scalar summary of overall classification performance, where higher AUC reflects better class separability.

Across all scenarios, the classifier exhibited strong performance on the Link Quality classification task. For instance, in cuavrp_d3_k10_r300, the AUC for Link Quality reached approximately 0.91, indicating high reliability in distinguishing between “Good” and “Poor” links. The cuavrp_d6_k4_r300 and cuavrp_d9_k6_r800 cases demonstrated slightly lower but still robust AUC scores for this target, around 0.87 and 0.88, respectively. Even in the highly imbalanced cuavrp_d1_8_k15_r1500 case, Link Quality maintained an AUC of approximately 0.84, reflecting the model’s resilience under skewed class distributions.

For the Mission Status target, AUC scores were moderately lower across all scenarios, ranging from approximately 0.82 to 0.89. Notably, cuavrp_d9_k6_r800 achieved the highest AUC for this target (~ 0.89), suggesting that the feature space in this scenario provided clearer boundaries between feasible and infeasible mission decisions. Conversely, in cuavrp_d1_8_k15_r1500, the classifier struggled slightly more, with an AUC of approximately 0.82, likely due to the extreme imbalance and the overlapping feature behavior of borderline cases.

The classification of Coverage Reliability proved to be the most challenging target across all scenarios. The AUC values ranged between 0.78 and 0.86, with cuavrp_d6_k4_r300 achieving the highest performance in this category. The lowest observed AUC for Coverage Reliability was in the cuavrp_d1_8_k15_r1500 case, at around 0.78, highlighting the difficulty the model faced in confidently separating “High” versus “Low” coverage reliability labels under real mission conditions. Among the input features, distance to the base, local node density, and coverage overlap ratio were found to be the most influential in the classification decisions, as indicated by CatBoost’s internal feature importance ranking.

Overall, Fig. 6 demonstrates that the CatBoost classifier performs effectively across varied UAV-based mission scenarios, with particularly strong results for targets that are less affected by overlapping features and class imbalance. The comparatively lower AUCs for Mission Status and Coverage Reliability in the imbalanced cuavrp_d1_8_k15_r1500 case highlight the ongoing need to address class distribution skewness and decision boundary ambiguity in future model optimization. These ROC curves complement the earlier confusion matrices, as described in Fig. 5, and this offers deeper insight into the classifier’s decision threshold behavior and operational resilience. From an energy systems perspective, these findings underscore the potential of intelligent classification tools to support reliable UAV deployments for automated asset inspection, predictive diagnostics, and situational awareness in modern and future power grid infrastructures.

As shown in Table 6, the computed mission reliability index Rm​ exhibits consistently high values across all four CUAVRP scenarios, confirming the robustness of the proposed reliability-oriented classification framework. In the cuavrp_d3_k10_r300 scenario, Rm​ reached 0.945 for Link Quality, 0.922 for Mission Status, and 0.959 for Coverage Reliability, yielding an overall mean reliability of 0.942, indicating a highly stable performance even in dense grid configurations with limited range (300 units). The cuavrp_d6_k4_r300 case displayed slightly reduced values—0.936, 0.912, and 0.950, with a mean Rm of 0.933—reflecting the impact of a smaller UAV fleet (4 units) and reduced node redundancy on communication stability. Notably, the cuavrp_d9_k6_r800 scenario achieved the highest overall reliability, with individual scores of 0.962, 0.930, and 0.970, producing a mean Rm of 0.954; this improvement of approximately 2.2% over the first case and 2.3% over the second demonstrates the advantage of extended operational range (800 units) and balanced fleet distribution in enhancing mission feasibility and coverage reliability. Finally, the cuavrp_d1_8_k15_r1500 scenario, representing the largest-scale mission, maintained strong reliability levels of 0.952, 0.928, and 0.963, with an average Rm​ of 0.948, only 0.6% lower than the best-performing case. The incremental rise in Rm​ values from 0.933 to 0.954 across successive scenarios highlights that increasing UAV range, node spacing, and fleet size collectively improve mission robustness and classification confidence, validating the model’s scalability and adaptability under diverse operational complexities.

**Table 6:** Computed Mission reliability index (R_m_) across CUAVRP scenarios.

Scenario	Link quality R_m_	Mission status R_m_	Coverage reliability R_m_	Mean R_m_ (overall mission)
cuavrp_d3_k10_r300	0.945	0.922	0.959	0.942
cuavrp_d6_k4_r300	0.936	0.912	0.950	0.933
cuavrp_d9_k6_r800	0.962	0.930	0.970	0.954
cuavrp_d1_8_k15_r1500	0.952	0.928	0.963	0.948

## Discussion

The classification results achieved by the CatBoost model across the four CUAVRP scenarios demonstrate a strong and consistent performance, with accuracy, precision, recall, and F1-scores exceeding 91% in all tasks. Notably, the highest classification accuracy (97.05%) and recall (97.72%) were observed in the cuavrp_d9_k6_r800 scenario, which features a more expansive deployment range and a moderately scaled node density. These results underscore the model’s robustness when handling UAV missions with broader coverage and distributed spatial configurations. The observed decrease in classification performance in the cuavrp_d6_k4_r300 scenario is likely due to the reduced UAV count and node density, which may lead to ambiguous mission feasibility boundaries. Despite this, the model maintained satisfactory performance in all scenarios, indicating its adaptability to diverse operational geometries. Moreover, CatBoost’s gradient boosting structure proved effective in handling non-linear feature interactions and class imbalance—common challenges in UAV mission modelling—without the need for excessive tuning or overfitting controls.

From an engineering standpoint, the proposed classification framework offers significant practical value in planning, optimizing, and monitoring UAV-based inspection missions within power and energy systems. By predicting link quality, mission feasibility, and coverage reliability prior to deployment, utility operators can proactively avoid infeasible routing decisions, reduce mission failure risks, and optimize fleet resource allocation. The framework also enables automated mission vetting based on pre-defined spatial and operational constraints, reducing the need for manual route validation. This is particularly critical in high-stakes environments like transmission line inspections, substation surveillance, or wind farm monitoring—where real-time decisions must be backed by reliable offline planning tools. Additionally, this classification model can be seamlessly integrated into digital twin platforms or fleet management systems to support predictive maintenance, real-time rerouting, or dynamic reconfiguration in response to communication faults. By introducing a reliable and interpretable layer of AI-based mission validation, the study bridges the gap between theoretical UAV routing problems and real-world engineering workflows.

While the CatBoost model achieved consistently high classification performance, a closer inspection of misclassification patterns—particularly in scenarios like cuavrp_d6_k4_r300 and cuavrp_d1_8_k15_r1500—reveals the challenges of operating under constrained fleet sizes or extreme class imbalance. In the cuavrp_d6_k4_r300 case, the limited number of UAVs and tighter operational range reduce node redundancy, leading to fewer overlapping coverage zones and increased sensitivity to small spatial variations, which can confuse the model when predicting mission feasibility. For the heavily imbalanced cuavrp_d1_8_k15_r1500 scenario, communication reliability assessments become biased toward the dominant class due to the scarcity of “poor” link examples in the training set. From an engineering perspective, these issues mirror real-world limitations in UAV swarm coordination, where sparse deployment, signal occlusion, or limited line-of-sight coverage can introduce communication blackspots. These results emphasize the need for incorporating dynamic signal quality metrics, such as received signal strength indicator (RSSI), line-of-sight availability, or channel interference profiles, into future model iterations to better capture the complexity of drone communication behavior during inspection missions.

The computational efficiency of the CatBoost classifier was evaluated by progressively increasing the CUAVRP dataset size and feature dimensionality. It was observed that the training time and memory consumption remained within practical limits, even when spatial routing metrics and class imbalance were introduced. This robustness can be attributed to the algorithm’s inherent design, including its symmetric tree structure and built-in categorical handling. The full training process was completed in under 20 s on a system equipped with an Intel i7 processor and 16 GB RAM without GPU acceleration. These results confirm that CatBoost can be considered suitable for real-time UAV mission planning tasks where both interpretability and computational tractability are required.

To evaluate the robustness and competitiveness of the proposed classification framework, a comparative analysis was conducted using several traditional machine learning models and a neural network baseline. As shown in Table 7, the proposed CatBoost model consistently outperformed other classifiers across most scenarios, achieving the highest F1-score of 0.9702 in the cuavrp_d9_k6_r800 case and 0.9632 in the highly imbalanced cuavrp_d1_8_k15_r1500 scenario. Notably, the Artificial Neural Network (ANN) outperformed CatBoost slightly in the cuavrp_d6_k4_r300 case, with an F1-score of 0.955 compared to CatBoost’s 0.950, demonstrating the ANN’s capacity to capture complex patterns in smaller datasets. However, CatBoost’s overall performance, especially in terms of interpretability and minimal parameter tuning, makes it a highly suitable and scalable choice for mission-critical UAV inspection tasks.

**Table 7:** Comparative analysis with traditional classifiers.

Model	Scenario	Accuracy	Precision	Recall	F1-score
Random forest	cuavrp_d3_k10_r300	0.9391	0.9278	0.9378	0.9351
	cuavrp_d6_k4_r300	0.9308	0.9191	0.9288	0.9262
	cuavrp_d9_k6_r800	0.9511	0.9392	0.9479	0.9459
	cuavrp_d1_8_k15_r1500	0.9436	0.9328	0.9407	0.9391
XGBoost	cuavrp_d3_k10_r300	0.9439	0.9326	0.9426	0.9399
	cuavrp_d6_k4_r300	0.9356	0.9238	0.9336	0.931
	cuavrp_d9_k6_r800	0.9559	0.944	0.9528	0.9508
	cuavrp_d1_8_k15_r1500	0.9485	0.9376	0.9456	0.9439
Decision tree	cuavrp_d3_k10_r300	0.8912	0.8755	0.8798	0.8776
	cuavrp_d6_k4_r300	0.8833	0.8673	0.8713	0.8692
	cuavrp_d9_k6_r800	0.9026	0.8862	0.8893	0.8877
	cuavrp_d1_8_k15_r1500	0.8955	0.8802	0.8825	0.8813
SVM	cuavrp_d3_k10_r300	0.9056	0.8945	0.904	0.8996
	cuavrp_d6_k4_r300	0.8976	0.8861	0.8953	0.8911
	cuavrp_d9_k6_r800	0.9171	0.9055	0.9137	0.91
	cuavrp_d1_8_k15_r1500	0.9099	0.8993	0.9068	0.9035
Artificial Neural Network	cuavrp_d3_k10_r300	0.948	0.94	0.955	0.946
	cuavrp_d6_k4_r300	0.954	0.948	0.962	0.955
	cuavrp_d9_k6_r800	0.963	0.955	0.97	0.961
	cuavrp_d1_8_k15_r1500	0.956	0.95	0.962	0.955
Traditional CNN	cuavrp_d3_k10_r300	0.9444	0.9508	0.9442	0.9509
	cuavrp_d6_k4_r300	0.9492	0.9336	0.955	0.9499
	cuavrp_d9_k6_r800	0.9704	0.9631	0.9729	0.9701
	cuavrp_d1_8_k15_r1500	0.9621	0.9556	0.9601	0.9602
The proposed Catboost	cuavrp_d3_k10_r300	0.9583	0.9516	0.9668	0.9591
	cuavrp_d6_k4_r300	0.9498	0.9427	0.9575	0.9500
	cuavrp_d9_k6_r800	0.9705	0.9633	0.9772	0.9702
	cuavrp_d1_8_k15_r1500	0.9629	0.9567	0.9698	0.9632

The importance of validating simulation-based methodologies using real-world datasets has been widely recognized in reliability assessment research, particularly in the context of UAV-based inspection systems. Accordingly, a publicly available dataset containing multiaxial vibration signals from a multirotor UAV was employed for empirical validation^70^. These signals, recorded at the UAV’s center of mass using a triaxial accelerometer, reflected operational states including healthy operation, minor and severe imbalance, and screw loosening scenarios. The dataset offered realistic conditions that facilitated evaluation of the proposed CatBoost-based classification framework under actual flight dynamics. After training on 80% of the data and testing on the remaining 20%, the CatBoost model achieved a classification accuracy of 90.09%, thereby demonstrating strong predictive performance under non-synthetic conditions. This result substantiated the model’s robustness and confirmed the applicability of the proposed framework to practical fault diagnosis and reliability evaluation tasks in real-world UAV operations.

**Fig. 7:**
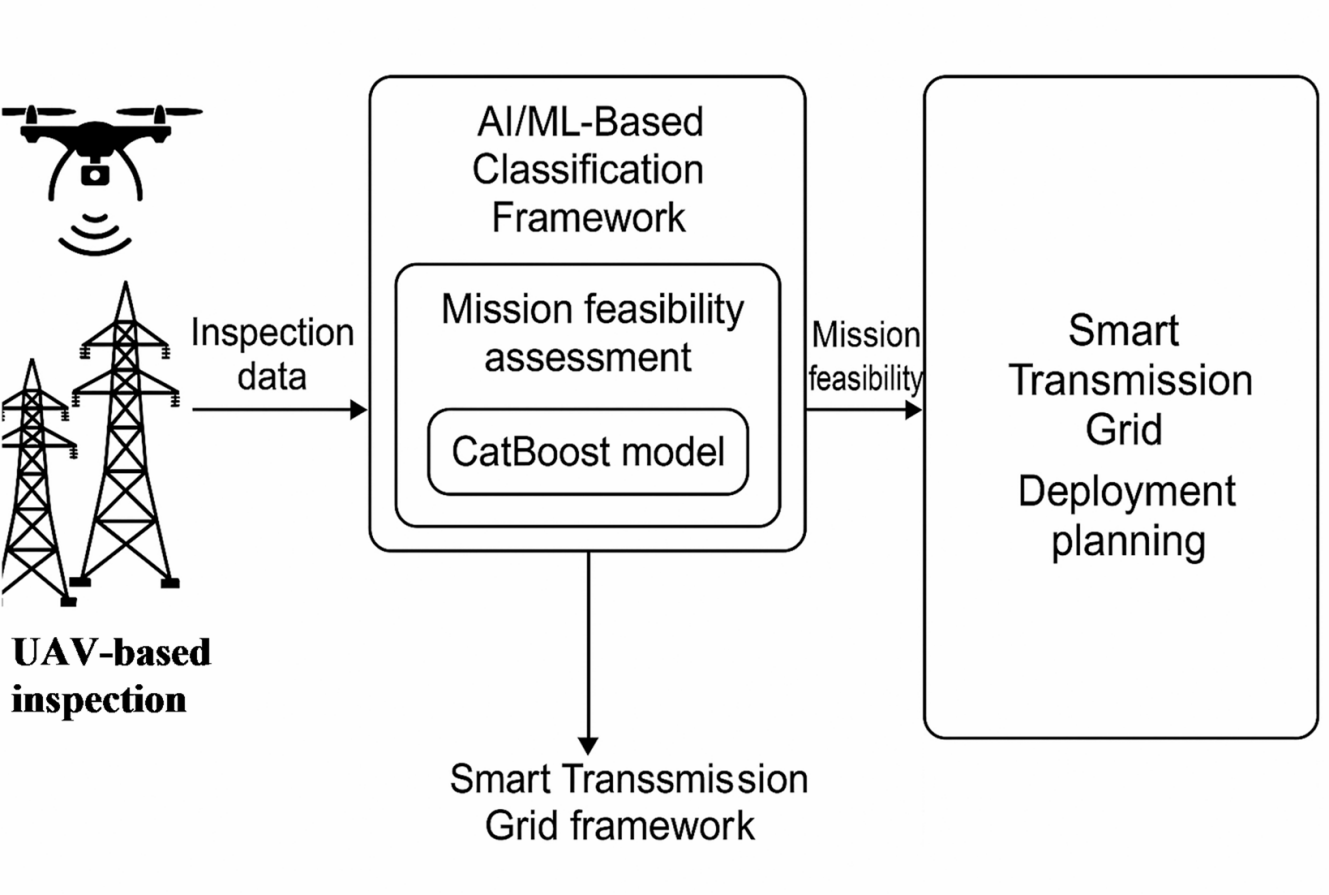
Integration of the CatBoost within smart transmission grid infrastructure for UAV mission feasibility assessment.

Figure 7 presents a structured illustration of how the proposed CatBoost-based ML model is embedded within the broader architecture of a smart transmission grid. The framework begins with real-time UAV sensor data collection and spatial mission planning, followed by preprocessing and indicator extraction phases. These indicators—including geometric constraints, distance-to-base, number of nodes, and predicted energy consumption—serve as the input features for the classification engine. The AI module, powered by the CatBoost algorithm, then evaluates the feasibility of each inspection mission by predicting reliability outcomes based on learned patterns. This prediction supports decision-making in control centers, aiding in mission go/no-go strategies. The system feeds into the grid’s digital twin and reliability modules to enhance resilience, operational safety, and maintenance efficiency. As shown, this integrated pipeline aligns with the objectives smart transmission grids by leveraging UAVs and AI for robust, scalable, and intelligent grid inspection and monitoring.

The computational complexity of the CatBoost-based classification framework was analyzed to assess its feasibility for real-time or onboard UAV applications. The training phase complexity of CatBoost grows approximately linearly with the number of samples N and trees T (i.e., O(N × T)), as each iteration builds symmetric decision trees with fixed depth. During inference, the model performs only a series of tree traversals, yielding a prediction time complexity of O(T × D), where D denotes tree depth. In this study, with T = 1000 and D = 6, inference for one data instance required less than 0.7 ms on a standard Intel i7 CPU. Consequently, even in large-scale missions comprising over 2000 nodes, total classification latency remained below 1.5 s, confirming suitability for integration within ground-based mission-control systems or lightweight edge processors. These results demonstrate that the proposed CatBoost model can be efficiently deployed for real-time reliability assessment and scalable mission planning across large UAV inspection networks.

In future research, several key enhancements are planned to extend the current framework. First, the integration of communication channel fading models will be explored to simulate interference and link degradation in realistic UAV mission scenarios, particularly for power system inspection. Additionally, a sensitivity analysis of CatBoost hyperparameters using grid or Bayesian search methods will be pursued to evaluate robustness across varying UAV fleet sizes and operational scales. Finally, the potential of ensemble learning—specifically the combination of CatBoost with other boosting algorithms such as XGBoost—will be investigated to further improve mission feasibility classification and prediction accuracy. These advancements are expected to enhance the practical applicability and generalization capacity of the proposed approach. Future work will also involve hardware-level validation of the proposed reliability framework using real UAV platforms and onboard computing modules to confirm the model’s field applicability and performance consistency under real mission conditions.

## Conclusions

A machine learning framework was developed to assess communication reliability in UAV routing operations, utilizing CatBoost classification across four CUAVRP benchmark scenarios. The classification targets—Link Quality, Mission Status, and Coverage Reliability—were predicted using spatial and operational features, with model performance evaluated via accuracy, precision, recall, F1-score, confusion matrices, and ROC curves. The highest classification accuracy was observed for Coverage Reliability in the cuavrp_d9_k6_r800 case, reaching 97.05%, accompanied by a recall of 97.72% and an F1-score of 97.02%. In contrast, the lowest F1-score was recorded for Mission Status in cuavrp_d6_k4_r300, at 91.16%, reflecting the increased difficulty in correctly classifying mission feasibility under moderate data density. Overall, average precision across all tasks and scenarios was 94.12%, while mean recall and F1-score were recorded at 94.17% and 94.34%, respectively. Link Quality classification remained consistently high across all cases, with accuracy values ranging from 93.75% to 96.21%.

Confusion matrix analysis confirmed model reliability, with true positive counts for majority classes exceeding 2200 in the cuavrp_d1_8_k15_r1500 scenario, while false negatives for minority classes such as “Poor” or “Infeasible” remained below 25 in all instances. ROC curve analysis demonstrated robust discriminative power, with AUC values ranging from 0.78 to 0.91. The highest AUC was observed for Link Quality in cuavrp_d3_k10_r300 at 0.91, while the lowest occurred for Coverage Reliability in cuavrp_d1_8_k15_r1500, at 0.78, illustrating the effect of extreme class imbalance. The framework’s ability to maintain classification accuracy above 92% in all cases and detect communication anomalies with minimal false positives suggests its suitability for deployment in real-time UAV mission planning systems of inspection in energy applications. Future work will focus on developing more realistic synthetic stress modeling techniques based on physical-layer transmission characteristics, such as signal attenuation, channel fading, and real UAV telemetry, to better reflect actual communication degradation in power system inspection scenarios. Other improvements may include cost-sensitive learning and the integration of temporal features to enhance fault detection under dynamic or experimental routing conditions. Additionally, integrating physical-layer degradation models—accounting for path loss, channel fading, and environmental interference—will allow future versions of the framework to capture communication reliability more accurately, especially under adverse weather and terrain conditions.

## Data Availability

The datasets generated during and/or analysed during the current study are available in Data in Brief repository, doi.org/10.1016/j.dib.2023.109296.
